# Three distinct cutaneous manifestations of Kikuchi-Fujimoto's disease and Adult-Onset Stills disease overlap syndrome

**DOI:** 10.1016/j.jdcr.2025.04.022

**Published:** 2025-05-02

**Authors:** An Jian Leung, Wei Wen Julian Lim, Lester Juay

**Affiliations:** a Division of Dermatology, Department of Medicine, National University Hospital, Singapore; b Division of Rheumatology, Department of Medicine, National University Hospital, Singapore

**Keywords:** acral pigmentation, Adult-Onset Stills disease, autoimmune, autoinflammatory syndromes, Kikuchi-Fujimoto’s disease

## Introduction

Kikuchi-Fujimoto's disease (KFD), also known as histiocytic necrotizing lymphadenitis, is a rare, self-limiting inflammatory disorder characterized by lymphadenopathy, elevated inflammatory markers, and histologic features of paracortical necrosis and histiocytic infiltrate on lymph node biopsy. Its co-existence with Adult-Onset Stills Disease (AOSD) has only been reported occasionally. AOSD is a systemic autoinflammatory disorder characterized by quotidian fevers, inflammatory arthritis, rash and neutrophilic leucocytosis, with typical cutaneous manifestations being a salmon-pink evanescent rash. Atypical varied cutaneous morphologies have been reported. We report an unusual case of KFD-AOSD overlap with 3 distinct cutaneous morphologies appearing during disease-flare, and a previously unreported acral pigmentation.

## Case details

**Fig 1:**
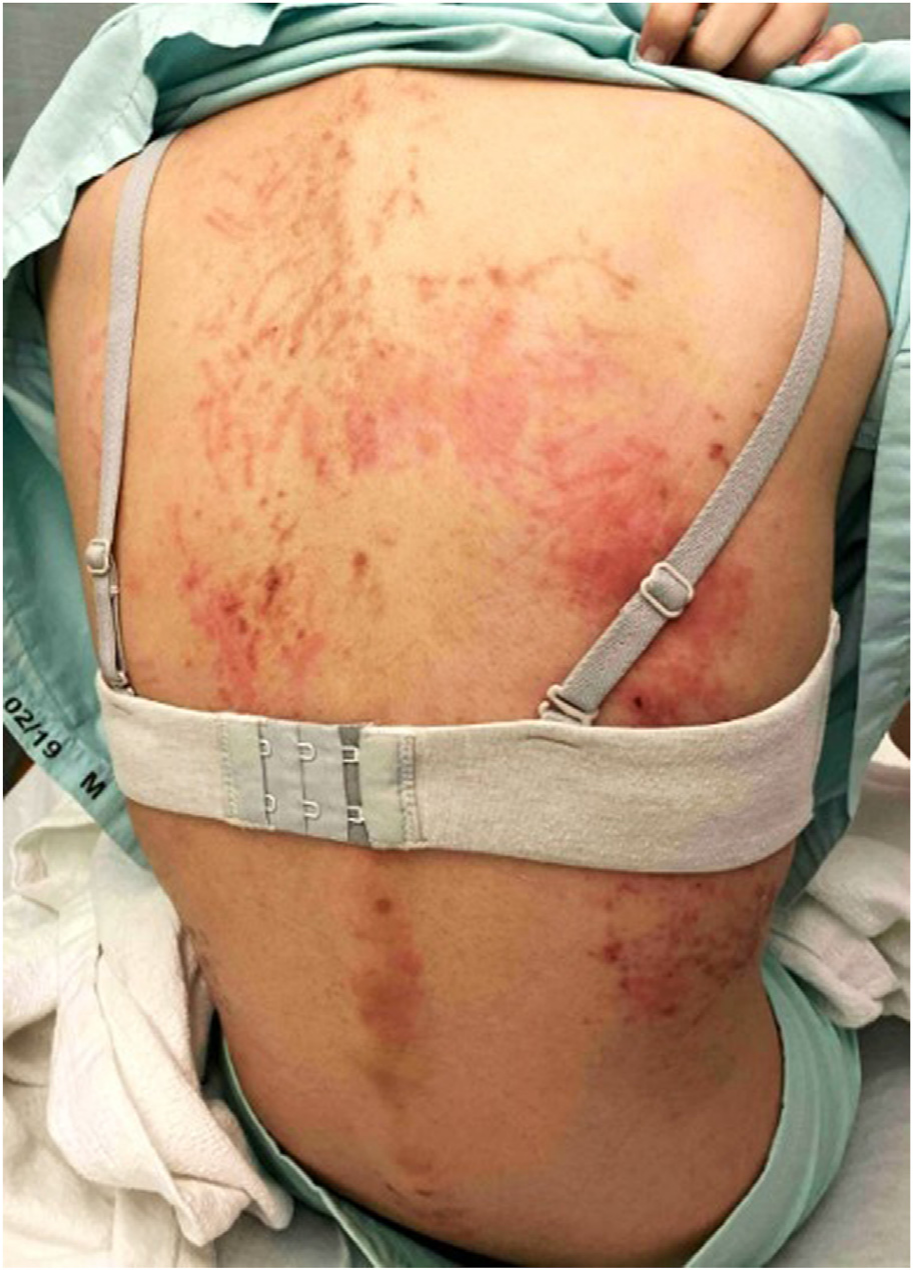
Flagellate dermatitis over the back occurring in early disease with salmon-pink evanescent plaques.

**Fig 2:**
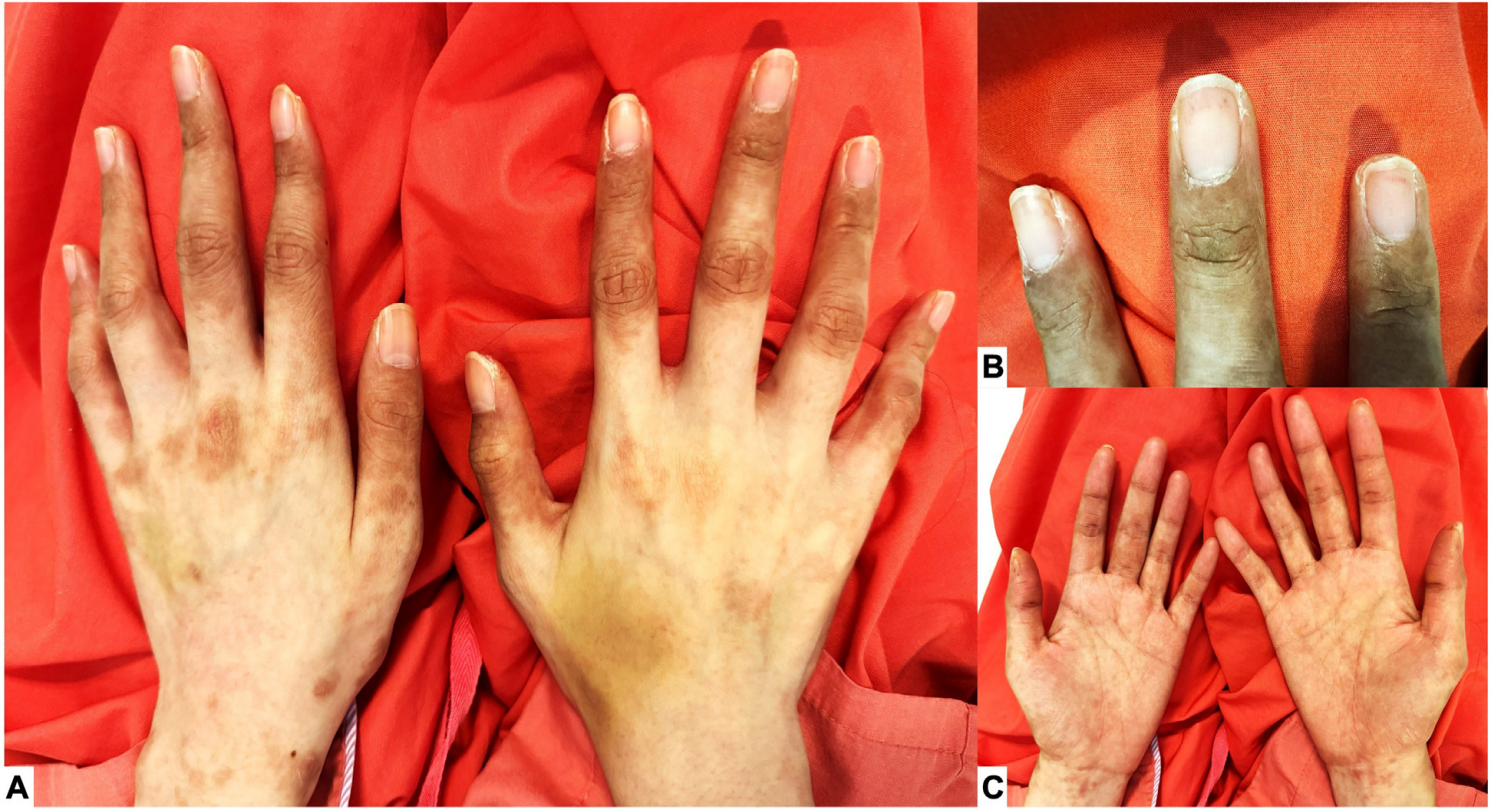
Acral hyperpigmentation 1 month into disease in the fingers affecting the dorsum of all digits (**A, B**) with knuckle pad involvement on the left hand (**A**). Palms (**C**) were largely spared. Healing with desquamation was subsequently observed.

**Fig 3:**
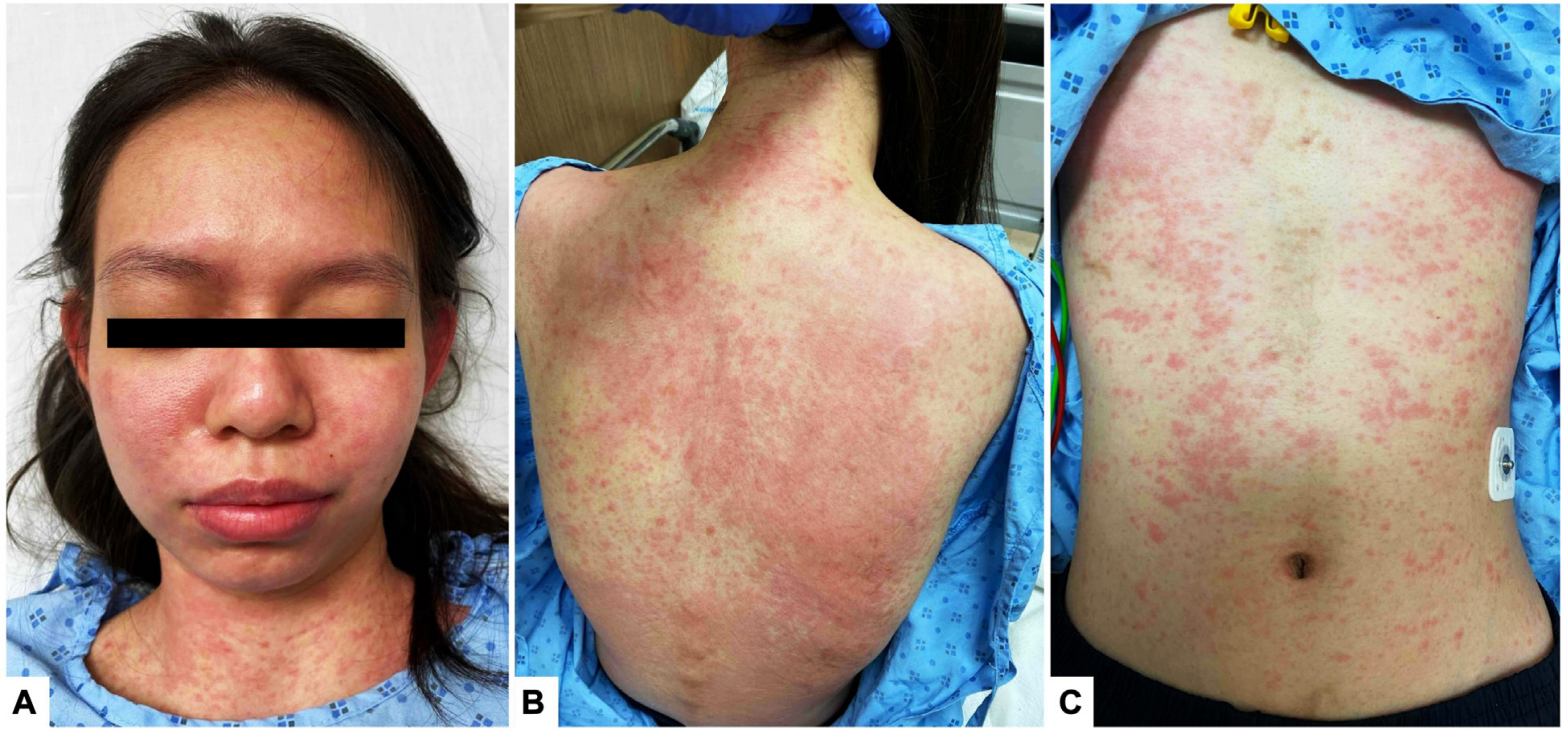
Generalized evanescent maculopapular exanthem over the face (**A**), neck and back (**B**), lower chest and abdomen (**C**) 2 months into disease with recurrence of flagellate dermatitis. Mucous membranes were spared.

A previously-well 22-year-old Chinese woman was treated for a 2-month history of daily high-spiking fevers, arthralgias, and fatigue. A physical examination revealed cervical and inguinal lymphadenopathy, and flagellate erythematous streaks over the back and flanks (Fig 1). She had no organomegaly, and mucous membranes were spared. Blood tests showed an elevated white blood cell count at 15.8 × 10^9^/L (80.6% neutrophils), elevated inflammatory markers; C-reactive protein of 183 mg/L, erythrocyte sedimentation rate of 58 mm/hr, procalcitonin of 63.5ug/L with hyperferritinemia (ferritin > 33 000 ug/L). She had significantly elevated liver enzymes (aspartate transaminase 658 U/L, alanine transaminase 412 U/L, alkaline phosphatase 422 U/L) and lactate dehydrogenase (2292 U/L). Serological markers were positive for anti-Sjögren's-syndrome-related antigen A Ro antibodies (41, positive >20 RU/mL), antismooth muscle antibody (119, positive >30 units) and a borderline antinuclear antibody at 1:80 with a homogeneous pattern. Rheumatoid factor was negative and serological markers for connective tissue and autoimmune disorders were unyielding. Schirmer’s test was equivocal (left eye 5 mm, right eye 7 mm). Chest radiograph revealed mild bilateral pleural effusions. An extensive infective evaluation was negative. A skin biopsy performed over the right flank during her initial presentation revealed perivascular dermatitis with neutrophils and eosinophils, mild interface changes with mild dermal mucin deposition, supporting the diagnosis of AOSD. Her index rash had healed 1 month into her disease, but she then developed new acral hyperpigmentation involving her distal fingers (Fig 2, *A*-*C*). These eventually resolved spontaneously with desquamation. Two months into her illness, a new evanescent maculopapular exanthem erupted (Fig 3, *A*-*C*) over her face, neck, torso, and back and limbs, with notable recurrence of flagellate dermatitis over the back. On all 3 cutaneous eruptions, she was noted to be in disease-flare with fevers, arthralgias and elevated inflammatory markers. Submandibular lymph node biopsy during her second flare showed reactive lymphohistiocytic proliferation, plasmacytoid dendritic cells, karyorrhexis and CD68+ crescenteric histiocytes. Neutrophils, granulomas, and malignant cells were notably absent. This suggested early-phase KFD. A liver biopsy was normal. The patient was treated with nonsteroidal anti-inflammatory drugs, oral and topical corticosteroids with rapid abatement of fevers and improvement of inflammatory markers and cutaneous lesions. At 3-month follow-up, she developed telogen effluvium, though previous cutaneous manifestations remained quiescent. At 6-month follow-up, she remained disease-free.

## Discussion

Cutaneous manifestations in AOSD/KFD overlap are varied. This case posed a diagnostic dilemma for 2 reasons – first, the unexpected evolution of her initial salmon-pink plaque and flagellate dermatitis to acral hyperpigmentation (a previously unreported association with AOSD or KFD) and second, the presence of 3 distinct rashes throughout her disease course.

The pathogenesis of AOSD involves an overactive innate immune reaction with excessive production of pro-inflammatory chemokines and cytokines with resultant systemic autoinflammation.^1^ The well-accepted diagnostic criteria outlined by Yamaguchi et al in 1992 confirms AOSD if at least 5 criteria are met, with at least 2 major criteria.^2^ Our patient met 4 major (fever, arthritis/arthralgia, typical rash and leucocytosis) and 2 minor (abnormal liver function tests and lymphadenopathy) criteria. Applying Fautrel’s 2002 criteria further confirms the diagnosis.^3^ However, the authors note that the borderline ANA positivity does not favour AOSD.^2^ KFD, first characterized among Asians in the 1970s, has no fixed diagnostic criteria though it is largely accepted that lymph node histological examination is crucial.^4^^,^^5^ It shares similar pathophysiological pathways as AOSD but has characteristic histological findings of T-cell and histiocyte-mediated immune hyper-reactivity.^4^^,^^5^ In our case, lymph node biopsy findings were suggestive of proliferative-stage KFD.^4^^,^^6^ On this basis and with the exclusion of other autoimmune, malignant, and infective etiologies, our patient was diagnosed with AOSD/KFD overlap disorder.

Classic criteria skin manifestations AOSD are an evanescent salmon-pink maculopapular exanthem.^1^^,^^2^ Flagellate dermatitis, though atypical, has been associated with systemic autoinflammatory disorders like AOSD with other implicated etiologies like Shiitake mushroom (*Lentinula edodes*) consumption, drug reaction from antineoplastic agents (bleomycin, docetaxel) or dermatomyositis. These causes were promptly excluded clinically and on serology.^3^ Atypical skin lesions reported in AOSD include erythematous, brown and violaceous papules and plaques, dermatomyositis-like rashes, lichenoid papules and prurigo pigmentosa-like eruptions.^1^, ^2^, ^3^ Epidermal changes like scales and crusting may be occasionally observed. They are commonly pruritic and tend to occur over the back, chest, abdomen and extensor surfaces. Rarely Koebner’s phenomenon, which accounts for a flagellate appearance, may be observed.^3^^,^^6^ Indeed, multiple varied cutaneous manifestations may appear simultaneous or sporadically over a disease course, as exemplified in our case. Acral hyperpigmentation is, however, morphologically distinct from previously reported cutaneous lesions associated with AOSD/KFD.^6^^,^^7^ It has a unique set of etiologies like nutritional deficiencies, autoimmune disorders (Addison’s disease), infections and depositional disorders, none of which were suspected in our case. It is pertinent to note the patient’s flagellate dermatitis exhibited characteristic waxing and waning with disease flares, while her acral pigmentation ran a monophasic clinical course.

K. A. Toribio in a 2014 retrospective study on AOSD/KFD highlighted that cutaneous lesions were observed in all overlap cases.^6^ This was peculiar since KFD alone had an estimated association of only 30% with cutaneous manifestations, while about 90% of AOSD patients exhibited skin manifestations. Toribio noted additionally that none of the AOSD/KFD skin lesions exhibited a temporal relationship with disease flare states, like the acral hyperpigmentation in our case.^6^^,^^8^^,^^9^ Of note, the patient’s 3 distinct cutaneous eruptions occurred during disease-flare, though flagellate dermatitis appeared only during the first and third flare.

We postulate that the occurrence of KFD with ongoing polycyclic AOSD accounted for acral hyperpigmentation. This is supported by its monophasic clinical course and its occurrence with lymphadenopathy, which is more attributable to KFD rather than AOSD based on existing knowledge.^10^ We cautiously hypothesize that systemic inflammation with acral micro-vasculopathy and dermal hemosiderin deposition may be a pathophysiological explanation for this unusual cutaneous morphology.

## Conclusion

The 3 distinct cutaneous morphologies observed in this case; (i) flagellate dermatitis, (ii) acral hyperpigmentation, and (iii) an evanescent maculopapular exanthem adds to the limited literature on cutaneous manifestations in AOSD/KFD overlap. Clinicians should remain vigilant for unusual cutaneous morphologies when treating systemic autoinflammatory syndromes and involve dermatologists early.

## Conflicts of interest

None disclosed.
